# Acute Loss of TrkB Signaling Impairs the Survival of Mature Retinal Ganglion Cells and Retinal Function

**DOI:** 10.1167/iovs.67.12.75

**Published:** 2026-10-28

**Authors:** Yuta Kitamura, Kazuhiko Namekata, Xiaoli Guo, Youichi Shinozaki, Sari Honda, Euido Nishijima, Takahiko Noro, Chikako Harada, Takayuki Harada

**Affiliations:** 1 Visual Research Project, Tokyo Metropolitan Institute of Medical Science, Tokyo, Japan; 2 Department of Ophthalmology and Visual Science, Chiba University Graduate School of Medicine, Chiba, Japan; 3 Department of Ophthalmology, Juntendo University School of Medicine, Tokyo, Japan; 4 Department of Ophthalmology, The Jikei University School of Medicine, Tokyo, Japan

**Keywords:** retinal ganglion cells, TrkB, BDNF, glaucoma, neuroprotection, gene therapy

## Abstract

**Purpose:**

To determine whether acute loss of tropomyosin receptor kinase B (TrkB) signaling in mature retinal ganglion cells (RGCs) induces RGC degeneration and retinal functional impairment in the adult mouse retina.

**Methods:**

TrkB deletion was induced in adult TrkB^flox/flox^ mice by intravitreal administration of adeno-associated virus serotype 2 (AAV2)-Cre. AAV2-Cre tropism was examined using tdTomato reporter mice and retinal cell markers. TrkB, RNA-binding protein with multiple splicing (RBPMS), calretinin, glutamine synthetase, and recoverin protein levels were assessed by western blotting. RGC survival was evaluated by RBPMS immunostaining of retinal flatmounts. Retinal function was assessed by analyzing the second-order kernel of multifocal electroretinography. Acute TrkB deletion was compared with developmental TrkB deletion in TrkB^flox/flox^;c-kit-Cre mice.

**Results:**

AAV2-Cre predominantly transduced RBPMS-positive RGCs, with little overlap with calretinin-positive amacrine cells, glutamine synthetase–positive Müller glia, or recoverin-positive photoreceptors. AAV2-Cre administration at 7 weeks of age reduced TrkB protein levels. RBPMS protein levels and RBPMS-positive RGC density were significantly decreased 5 weeks after AAV2-Cre administration at 12 weeks of age, whereas calretinin, glutamine synthetase, and recoverin protein levels were unchanged. Retinal responses of the second-order kernel were also significantly reduced. In contrast, RGC density was not significantly altered in TrkB^flox/flox^;c-kit-Cre mice at 12 weeks of age.

**Conclusions:**

Acute TrkB deletion in the adult retina induces RGC degeneration and impairs retinal function, whereas no significant reduction in RGC density was detected following developmental TrkB deletion at 12 weeks of age. These findings indicate that endogenous TrkB signaling is required for mature RGC maintenance and suggest that reduced TrkB signaling may contribute to glaucomatous RGC degeneration.

Glaucoma is one of the leading causes of irreversible vision loss worldwide and is characterized by progressive degeneration of retinal ganglion cells (RGCs) and their axons. Elevated intraocular pressure is a major risk factor for glaucoma, and current pharmacological treatments are primarily aimed at lowering intraocular pressure. However, glaucomatous neurodegeneration is a multifactorial process. In addition to mechanical stress associated with elevated intraocular pressure, aging,^1^ impaired blood flow,^2^ oxidative stress,^3^^–^^6^ glutamate excitotoxicity,^7^^–^^9^ and reduced neurotrophic support^10^^–^^12^ have been implicated in the pathogenesis and progression of glaucoma. These observations suggest that direct neuroprotective strategies targeting RGC survival pathways may complement conventional pressure-lowering therapies.

Brain-derived neurotrophic factor (BDNF) and its high-affinity receptor, tropomyosin receptor kinase B (TrkB), constitute one of the major neurotrophic signaling pathways that support neuronal survival and function in the central nervous system. In the retina, BDNF–TrkB signaling has been shown to promote RGC survival under physiological and pathological conditions.^13^^,^^14^ Reduced expression of BDNF and TrkB has been reported in association with aging and glaucoma,^15^ and decreased BDNF–TrkB signaling in the retina and optic nerve head may contribute to the vulnerability of RGCs in glaucomatous neurodegeneration.^10^ These findings raise the possibility that loss of TrkB signaling is not merely a consequence of RGC degeneration but may also directly participate in the initiation or progression of RGC loss.

We previously generated mice in which TrkB was deleted in RGCs and amacrine cells from early developmental stages using a c-kit-Cre line.^14^ In these mice, retinal structure was largely preserved at 3 months of age, although progressive RGC loss became detectable at 6 months of age. These observations suggested that developmental reduction of TrkB does not severely impair retinal formation but may increase susceptibility to age-dependent RGC degeneration. However, developmental deletion models may involve compensatory mechanisms that mask the requirement for TrkB in mature RGCs. In human glaucoma, TrkB signaling is more likely to decline during adulthood rather than being absent from development.^16^^,^^17^ Therefore, it remains unclear whether acute loss of TrkB in mature RGCs is sufficient to induce RGC degeneration and functional impairment.

In the present study, we investigated the role of TrkB signaling in the maintenance of mature RGCs by inducing acute TrkB deletion in the adult mouse retina. To this end, we used intravitreal administration of adeno-associated virus serotype 2 (AAV2)-Cre in TrkB^flox/flox^ mice and examined its effects on TrkB levels, RGC survival, and retinal function. We also compared the effects of acute TrkB deletion in adulthood with those of developmental TrkB deletion. This approach allowed us to evaluate whether TrkB signaling is required for the maintenance of mature RGCs and to distinguish its role in adult RGC survival from its contribution during retinal development.

## Materials and Methods

### Animals

Male mice were used in all experiments. Experiments were initiated at 10 and 7 weeks of age in tdTomato reporter mice and TrkB^flox/flox^ mice, respectively, whereas tissues from TrkB^flox/flox^;c-kit-Cre mice were collected at 12 weeks of age. The TrkB^flox/flox^ mice and TrkB^flox/flox^;c-kit-Cre mice were described previously.^14^^,^^18^ Mice were maintained under standard laboratory conditions with free access to food and water. All animal procedures were performed in accordance with the ARVO Statement for the Use of Animals in Ophthalmic and Vision Research and the Tokyo Metropolitan Institute of Medical Science Guidelines for the Care and Use of Animals. All animal experiments were approved by the Institutional Animal Care and Use Committee of the Tokyo Metropolitan Institute of Medical Science (approval no. 25-007).

### Preparation of AAV Vectors

AAV2 vectors were produced and purified as described previously.^18^ Briefly, human embryonic kidney 293 (HEK293) cells were transiently transfected with the AAV expression vector, pRC2-mi342, and pHelper plasmids (Takara Bio, Shiga, Japan) using Polyethylenimine HCl MAX (PEI MAX; Kyfora Bio, Horsham, PA, USA). The AAV expression vector used in this study contained a Cre recombinase expression cassette driven by the CAG promoter. Seventy-two hours after transfection, cells were harvested by scraping and subjected to three cycles of freeze–thawing. Cell debris was removed by centrifugation at 5000*g* for 20 minutes, and the supernatants were treated with Benzonase (200 U/mL; MilliporeSigma, Burlington, MA) in the presence of 5-mM MgCl_2_ at 37°C for 1 hour. The Benzonase-treated viral solution was purified using an iodixanol gradient. Purified AAV vectors were washed with Hanks’ Balanced Salt Solution and concentrated using VIVASPIN 20 centrifugal concentrators (Sartorius Stedim Lab, Göttingen, Germany) with a 100-kDa molecular weight cutoff. Viral titers were determined by quantitative polymerase chain reaction.

### Intravitreal Administration of AAV Vectors

Mice were anesthetized with a mixture of medetomidine (0.3 mg/kg body weight [bw]), midazolam (4.0 mg/kg bw), and butorphanol (5.0 mg/kg bw). Mice received intravitreal injections of AAV vectors under a surgical microscope with a microsyringe with a fine-gauge needle. A volume of 2 µL of AAV2-Cre (2.0 × 10^13^ vg/mL) was injected into the vitreous cavity of each eye. Care was taken to avoid injury to the lens. For analysis of AAV2-Cre tropism, AAV2-Cre was injected into tdTomato reporter mice at 10 weeks of age, and retinas were examined at 12 weeks of age, 2 weeks after injection. For acute TrkB deletion, AAV2-Cre was injected into TrkB^flox/flox^ mice at 7 weeks of age, and retinal samples were collected at 10 or 12 weeks of age, corresponding to 3 or 5 weeks after injection, respectively, as indicated in each experiment.

### Multifocal Electroretinography

Multifocal electroretinography (mfERG) was performed using a VERIS 6.0 system (Electro-Diagnostic Imaging, Milpitas, CA, USA) as described previously.^19^^,^^20^ Briefly, the visual stimulus consisted of seven hexagonal areas scaled with eccentricity. The stimulus array was displayed on a high-resolution black-and-white monitor driven at a frame rate of 100 Hz. The mfERG recordings were obtained before and 5 weeks after intravitreal AAV2-Cre administration in TrkB^flox/flox^ mice. We analyzed the amplitude of the second-order kernel, which reflects inner retinal function^21^ and is impaired in glaucoma patients.^22^ Data represent the average response over a 7-minute recording period for each eye.

### Immunostaining of Retinal Sections and Flatmounts

Mice were deeply anesthetized and perfused with 4% paraformaldehyde in 0.1-M phosphate buffer. Eyes were enucleated and postfixed in the same fixative for 2 hours. For retinal sections, eyes were cryoprotected in 30% sucrose in 0.1-M phosphate buffer for 24 hours, embedded in Tissue-Tek OCT Compound (Sakura Finetek Japan, Tokyo, Japan), and frozen. Retinal sections of 14-µm thickness were cut on a cryostat at −20°C.

For immunostaining, retinal sections were incubated with primary antibodies against RNA-binding protein with multiple splicing (RBPMS; 1:1000; Merck, Darmstadt, Germany), calretinin (1:1000; Proteintech, Rosemont, IL, USA), Sox9 (1:1000; Merck), glutamine synthetase (GS; 1:1000; Chemicon International, Temecula, CA, USA), and recoverin (1:1000; Merck), followed by incubation with appropriate Alexa Fluor 488– or Alexa Fluor 568–conjugated secondary antibodies (1:1000; Thermo Fisher Scientific, Waltham, MA, USA).

For retinal flatmount preparations, retinas were isolated from the eyecup and incubated for 2 hours in a blocking solution containing 5% horse serum and 1% Triton X-100 in PBS. The retinas were then incubated with anti-RBPMS antibody (1:1000; Merck) at 4°C for 24 hours. After washing with PBS, retinas were incubated with appropriate fluorescence-labeled secondary antibodies at 4°C for 24 hours. Images were obtained using an FV3000 confocal microscope (Olympus, Tokyo, Japan) or an All-in-One Fluorescence Microscope BZ-X800 (Keyence, Osaka, Japan).

### Quantification of RGC Density

RBPMS-positive RGCs were quantified in retinal flatmounts.^23^ Images were obtained from one central area, one middle area, and one peripheral area per quadrant of each retina. The central, middle, and peripheral areas were located 0.1, 0.8, and 1.5 mm from the optic disc, respectively. Each counting area was 0.04 mm^2^. RGCs were counted in the dorsal, ventral, nasal, and temporal quadrants, and the average RGC density was calculated as cells per square millimeter. For developmental TrkB deletion experiments, total RBPMS-positive RGC density was compared between TrkB^flox/flox^ control mice and TrkB^flox/flox^;c-kit-Cre mice at 12 weeks of age.

### Western Blot Analysis

Retinal samples were homogenized in lysis buffer and subjected to sodium dodecyl sulfate–polyacrylamide gel electrophoresis (SDS-PAGE) and immunoblot analysis. Membranes were incubated with primary antibodies against TrkB (1:1000; Cell Signaling Technology, Danvers, MA, USA), RBPMS (1:1000; Merck), calretinin (1:1000; Proteintech), GS (1:1000; Chemicon International), recoverin (1:1000; Merck), and β-actin (1:2000; BD Biosciences, Franklin Lakes, NJ, USA). After incubation with appropriate horseradish peroxidase–conjugated secondary antibodies, immunoreactive bands were detected using ECL Plus Western Blotting Detection Reagent (Nacalai Tesque, Kyoto, Japan) and visualized using a FUSION imaging system (Vilber, Collégien, France). Quantitative analysis was performed using ImageJ 2.14.0 (National Institutes of Health, Bethesda, MD, USA). Protein levels were normalized to β-actin.

### Statistical Analysis

Data are presented as mean ± SEM. Statistical analyses were performed using Prism 9.3.1 (GraphPad Software, Boston, MA, USA). Differences between two groups were analyzed using Welch's unpaired *t*-test or the Mann–Whitney *U* test. *P* < 0.05 was considered statistically significant.

## Results

### No Significant Reduction in RGC Density Is Detected Following Developmental TrkB Deletion at 12 Weeks of Age

To investigate effects of developmental TrkB deletion on RGC degeneration, RBPMS-positive RGCs were examined in TrkB^flox/flox^ control mice and TrkB^flox/flox^;c-kit-Cre mice at 12 weeks of age. Representative retinal flatmount images from the center, middle, and periphery of the retina showed comparable RBPMS-positive cell densities between the two groups (Fig. 1A). Quantitative analysis revealed no significant difference in total RGC density between TrkB^flox/flox^ control mice (3145 ± 162 cells/mm^2^) and TrkB^flox/flox^;c-kit-Cre mice (3267 ± 131 cells/mm^2^; *P* = 0.5932) (Fig. 1B). Although the sample size was limited (*n* = 3 mice per group), these findings suggest that developmental deletion of TrkB is not associated with substantial RGC degeneration at this age, consistent with our previous observations.^18^ Effects of acute TrkB deletion were then investigated.

**Figure 1.**
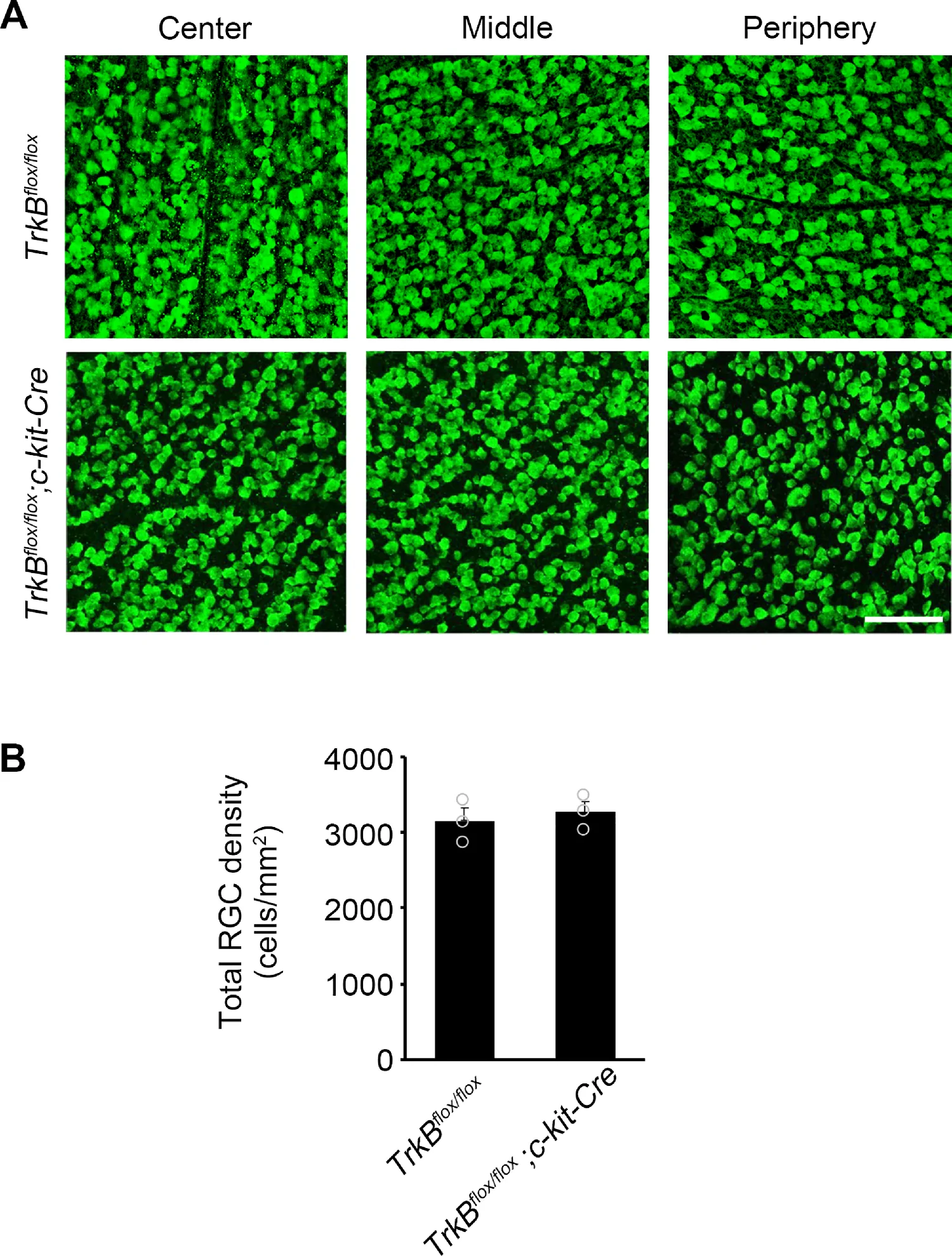
RGC density following developmental deletion of TrkB. (**A**) Representative images of RBPMS-immunostained retinal flatmounts from TrkB^flox/flox^ control mice and TrkB^flox/flox^;c-kit-Cre mice at 12 weeks of age. Images were acquired from the center, middle, and periphery of the retina. *Scale bar*: 50 µm. (**B**) Quantification of total RBPMS-positive RGC density. No significant difference in RGC density was observed between TrkB^flox/flox^ control mice and TrkB^flox/flox^;c-kit-Cre mice at 12 weeks of age. Data are presented as mean ± SEM. Statistical significance was determined using Welch's unpaired *t*-test (*n* = 3 eyes from 3 mice per group).

### AAV2-Cre Selectively Transduces RGCs After Intravitreal Administration

To induce TrkB deletion in adult RGCs, the cellular tropism of intravitreally administered AAV2-Cre was investigated in the retina. AAV2-Cre was injected into tdTomato reporter mice, and tdTomato expression was analyzed in retinal flatmounts and cross-sections. In retinal flatmount preparations, tdTomato-positive cells were widely distributed in the ganglion cell layer and showed extensive overlap with RBPMS-positive RGCs (Fig. 2A). In retinal cross-sections, tdTomato expression was predominantly detected in RBPMS-positive cells. By contrast, little overlap was observed between tdTomato and markers for other retinal cell types, including calretinin-positive amacrine cells, GS-positive Müller glia, and recoverin-positive photoreceptors (Fig. 2B). These results indicate that intravitreally administered AAV2-Cre preferentially transduces RGCs in the mouse retina.

**Figure 2.**
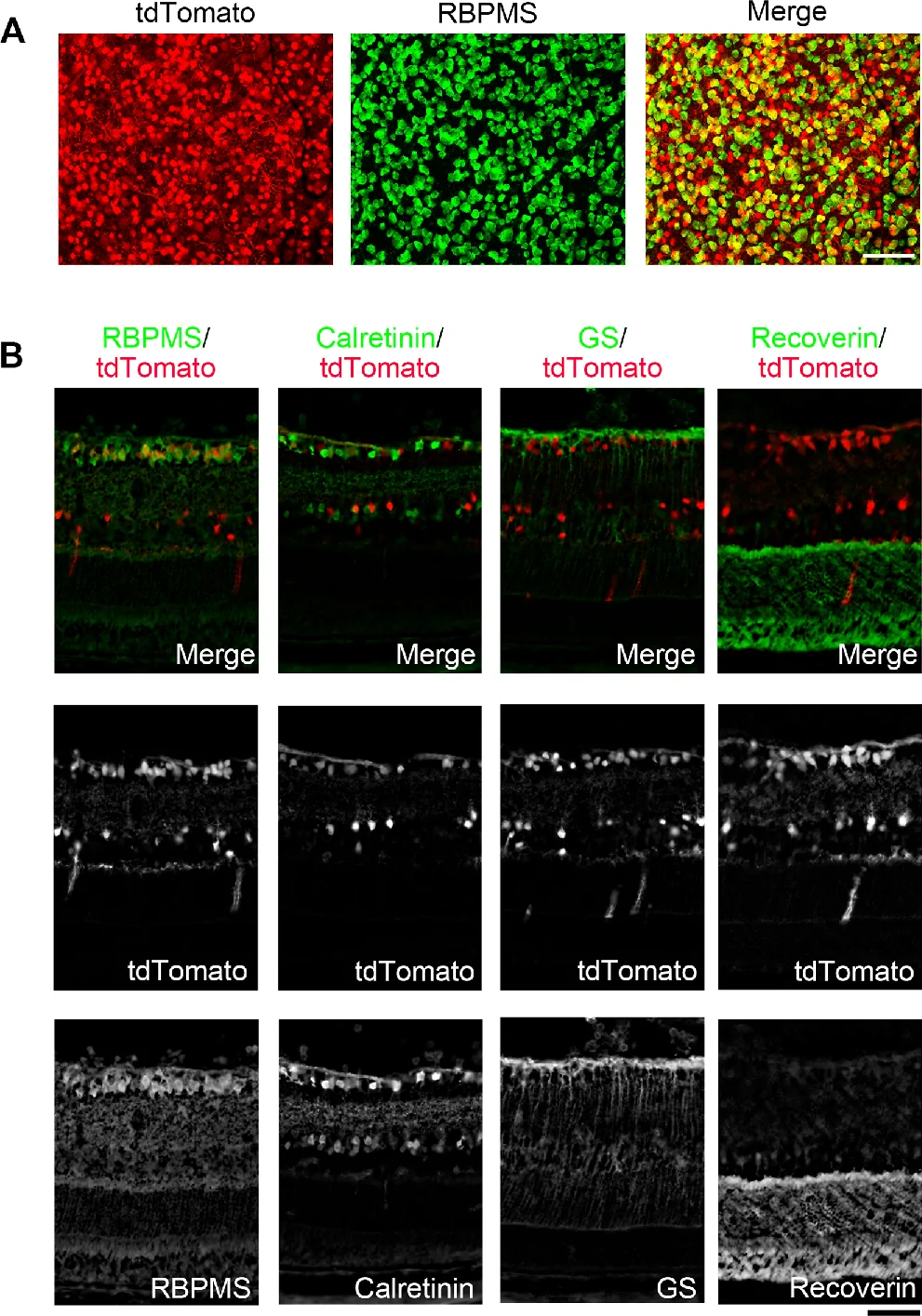
Selective transduction of retinal ganglion cells by intravitreally administered AAV2-Cre. (**A**) Representative retinal flatmount images from tdTomato reporter mice after intravitreal administration of AAV2-Cre. The tdTomato-positive cells were co-immunostained with RBPMS, a RGC marker. Merged images show extensive overlap between tdTomato and RBPMS, indicating efficient transduction of RGCs by AAV2-Cre. *Scale bar*: 100 µm. (**B**) Representative retinal cross-sections immunostained for RBPMS, calretinin, GS, and recoverin. The expression of tdTomato was predominantly observed in RBPMS-positive RGCs, whereas little overlap was detected with calretinin-positive amacrine cells, GS-positive Müller glia, or recoverin-positive photoreceptors. These results demonstrate that intravitreally administered AAV2-Cre selectively targets RGCs. *Scale bar*: 50 µm.

### Acute TrkB Deletion Reduces RBPMS Protein Levels in Adult TrkB^flox/flox^ Mice

To examine whether intravitreal administration of AAV2-Cre efficiently reduced TrkB protein levels, 7-week-old TrkB^flox/flox^ mice were used. Retinal lysates were prepared at 3 and 5 weeks after AAV2-Cre administration and analyzed by immunoblotting. TrkB protein levels were reduced after AAV2-Cre administration, consistent with efficient Cre-mediated deletion of TrkB in the retina (Figs. 3A–C, Supplementary Fig. S1). Quantitative analysis showed that normalized TrkB protein levels were significantly reduced from 1.00 ± 0.04 to 0.22 ± 0.07 at 3 weeks (*P* = 0.0015) and from 0.92 ± 0.15 to 0.22 ± 0.06 at 5 weeks (*P* = 0.0282) after AAV2-Cre administration. The protein levels of RBPMS, calretinin, GS, and recoverin were also examined. RBPMS protein levels were not significantly different at 3 weeks (1.00 ± 0.09 vs. 0.78 ± 0.07; *P* = 0.1491) but were significantly reduced at 5 weeks (0.93 ± 0.01 vs. 0.58 ± 0.03; *P* = 0.0032) after AAV2-Cre administration. In contrast, calretinin, GS and recoverin protein levels were not significantly altered (Figs. 3B, 3C).

**Figure 3.**
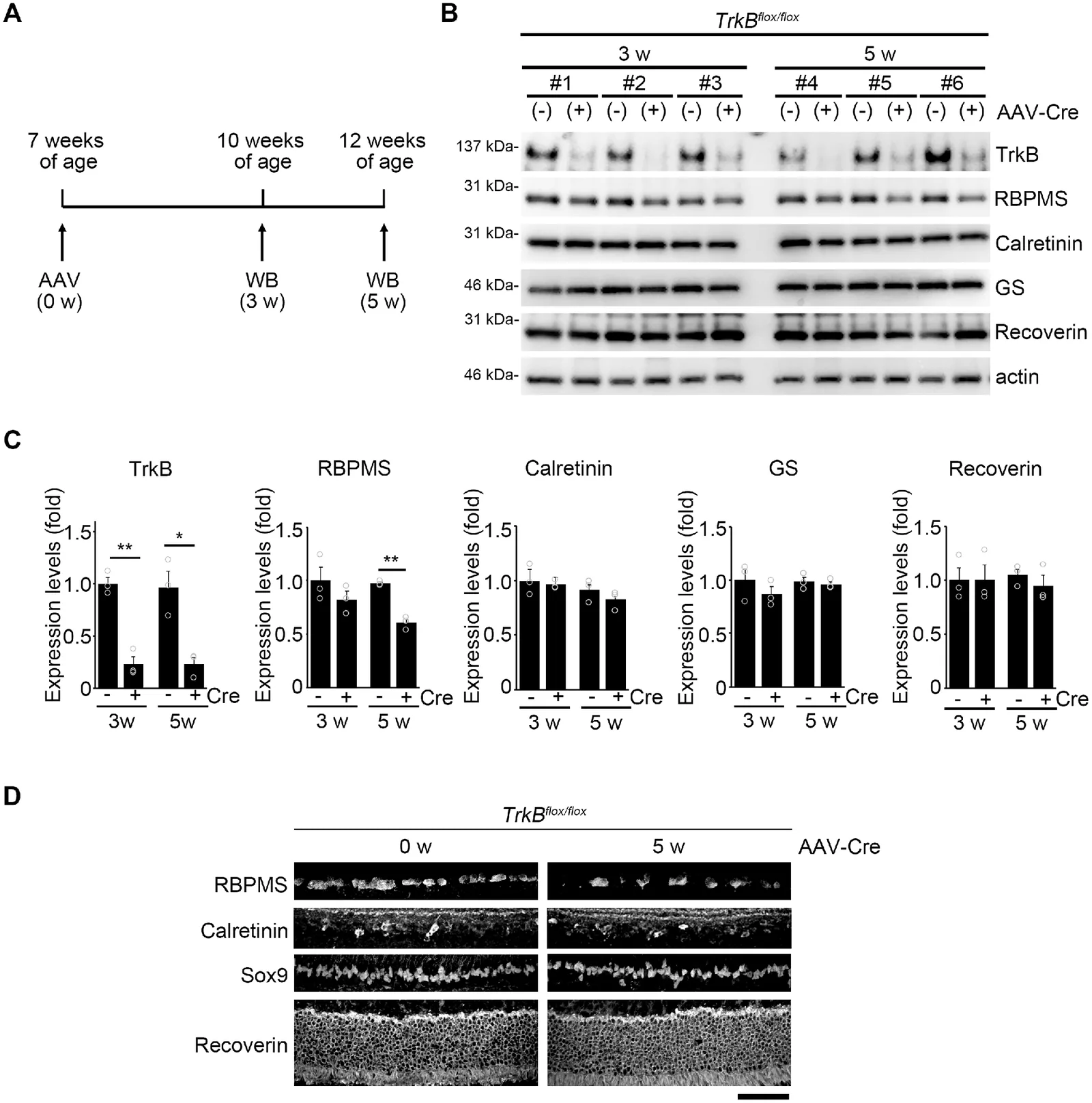
AAV2-Cre–mediated deletion of TrkB reduces RGC marker protein levels in TrkB^flox/flox^ mice. (**A**) Experimental schedule. AAV2-Cre was intravitreally administered to 7-week-old TrkB^flox/flox^ mice, and western blot analysis was performed 3 and 5 weeks after administration, at 10 and 12 weeks of age, respectively. (**B**) Representative immunoblots of retinal lysates collected from TrkB^flox/flox^ mice at 3 and 5 weeks after intravitreal administration of AAV2-Cre. Protein levels of TrkB, RBPMS, calretinin, GS, and recoverin were examined, with β-actin used as a loading control. (**C**) Quantification of TrkB, RBPMS, calretinin, GS, and recoverin protein levels normalized to β-actin. AAV2-Cre administration markedly reduced TrkB protein levels. RBPMS protein levels were significantly decreased at 5 weeks after AAV2-Cre administration, whereas calretinin, GS, and recoverin protein levels were not significantly altered. Data are presented as mean ± SEM. Statistical significance was determined using Welch's unpaired *t*-test (*n* = 3 eyes per group). *P < 0.05, **P < 0.01. (**D**) Representative retinal sections immunostained for RBPMS, calretinin, Sox9, and recoverin before AAV2-Cre administration and 5 weeks after administration. RBPMS immunoreactivity was reduced after TrkB deletion, whereas no obvious changes were observed in calretinin-positive amacrine cells, Sox9-positive Müller glia, or recoverin-positive photoreceptors. *Scale bar*: 50 µm.

Consistent with the immunoblot results, immunohistochemical analysis of retinal sections showed reduced RBPMS immunoreactivity 5 weeks after AAV2-Cre administration (Fig. 3D). No obvious morphological changes were observed in calretinin-positive amacrine cells, Sox9-positive Müller glia, or recoverin-positive photoreceptors. Together with the quantitative immunoblot analysis, these findings suggest that acute TrkB deletion preferentially affects RGCs, but the examined markers of other major retinal cell populations remained relatively unchanged.

### Acute TrkB Deletion Impairs Retinal Function Associated With RGC Degeneration

To determine the functional consequences of the reduction in RBPMS protein levels, retinal function was assessed by analyzing the second-order kernel of mfERG before and 5 weeks after AAV2-Cre administration (Figs. 4A, 4B). Representative response density maps and waveforms showed reduced retinal responses after AAV2-Cre–mediated TrkB deletion (Fig. 4B). Quantification of retinal responses of the second-order kernel demonstrated a significant reduction from 2.731 ± 0.145 before AAV2-Cre administration to 2.020 ± 0.156 at 5 weeks after administration (*P* = 0.0117) (Fig. 4C). These results indicate that acute TrkB deletion in the adult retina leads to retinal functional impairment.

**Figure 4.**
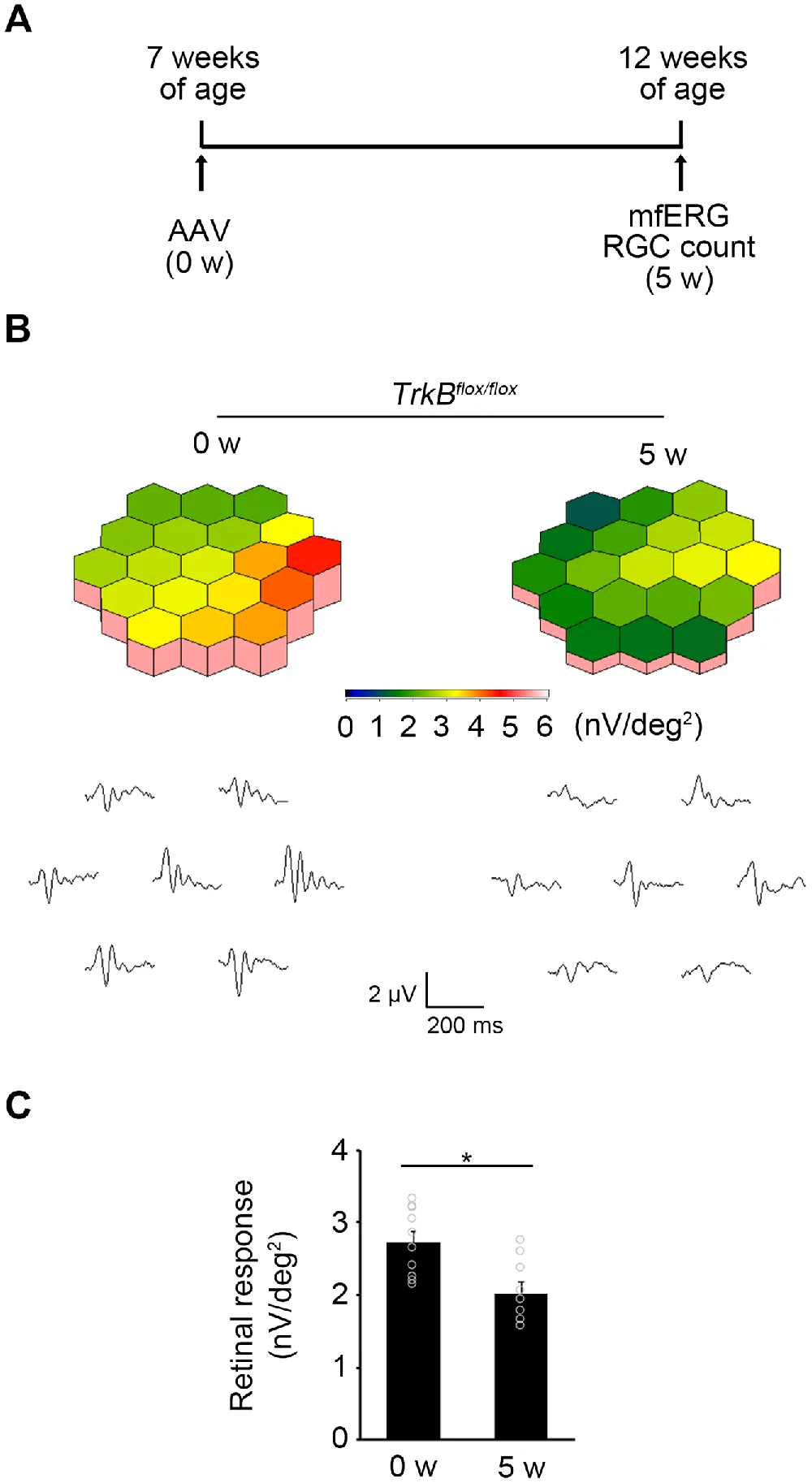
Acute deletion of TrkB impairs retinal function. (**A**) Experimental schedule. AAV2-Cre was intravitreally administered to 7-week-old TrkB^flox/flox^ mice, and mfERG analysis was performed 5 weeks after administration at 12 weeks of age. (**B**) Representative mfERG response density maps and waveforms of the second-order kernel at 0 weeks and 5 weeks after AAV2-Cre administration. (**C**) Quantification of retinal responses of the second-order kernel. The VERIS system allows the seven local second-order kernel responses to be displayed either as waveforms or as numerical amplitude values. For quantification, the numerical display was used, and the seven amplitude values automatically provided by the VERIS system were averaged to obtain a single retinal response value for each eye. Retinal responses were significantly reduced 5 weeks after AAV2-Cre administration. Data are presented as mean ± SEM. Statistical significance was determined using the Mann–Whitney U test (*n* = 12 eyes from 12 mice at 0 week; *n* = 8 eyes from 8 mice at 5 weeks). *P < 0.05.

### Acute TrkB Deletion in Mature RGCs Induces RGC loss

To determine whether the impairment of retinal function was accompanied by RGC loss, RBPMS-positive cells in retinal flatmounts were quantified. RBPMS-positive RGCs were examined in the center, middle, and periphery of the retina before AAV2-Cre administration and 5 weeks after administration. Representative flatmount images showed a clear reduction in RBPMS-positive cells in all retinal regions after AAV2-Cre–mediated TrkB deletion (Fig. 5A). Quantitative analysis confirmed that RGC density was significantly decreased at 5 weeks after AAV2-Cre administration in the center (3145 ± 233 vs. 1617 ± 378 cells/mm²; *P* = 0.0187), middle (3239 ± 58 vs. 1837 ± 341 cells/mm²; *P* = 0.0024), and periphery (2745 ± 107 vs. 1267 ± 318 cells/mm²; *P* = 0.0006) of the retina (Fig. 5B). These results indicate that acute TrkB deletion in the adult retina leads to RGC loss in addition to retinal functional impairment.

**Figure 5.**
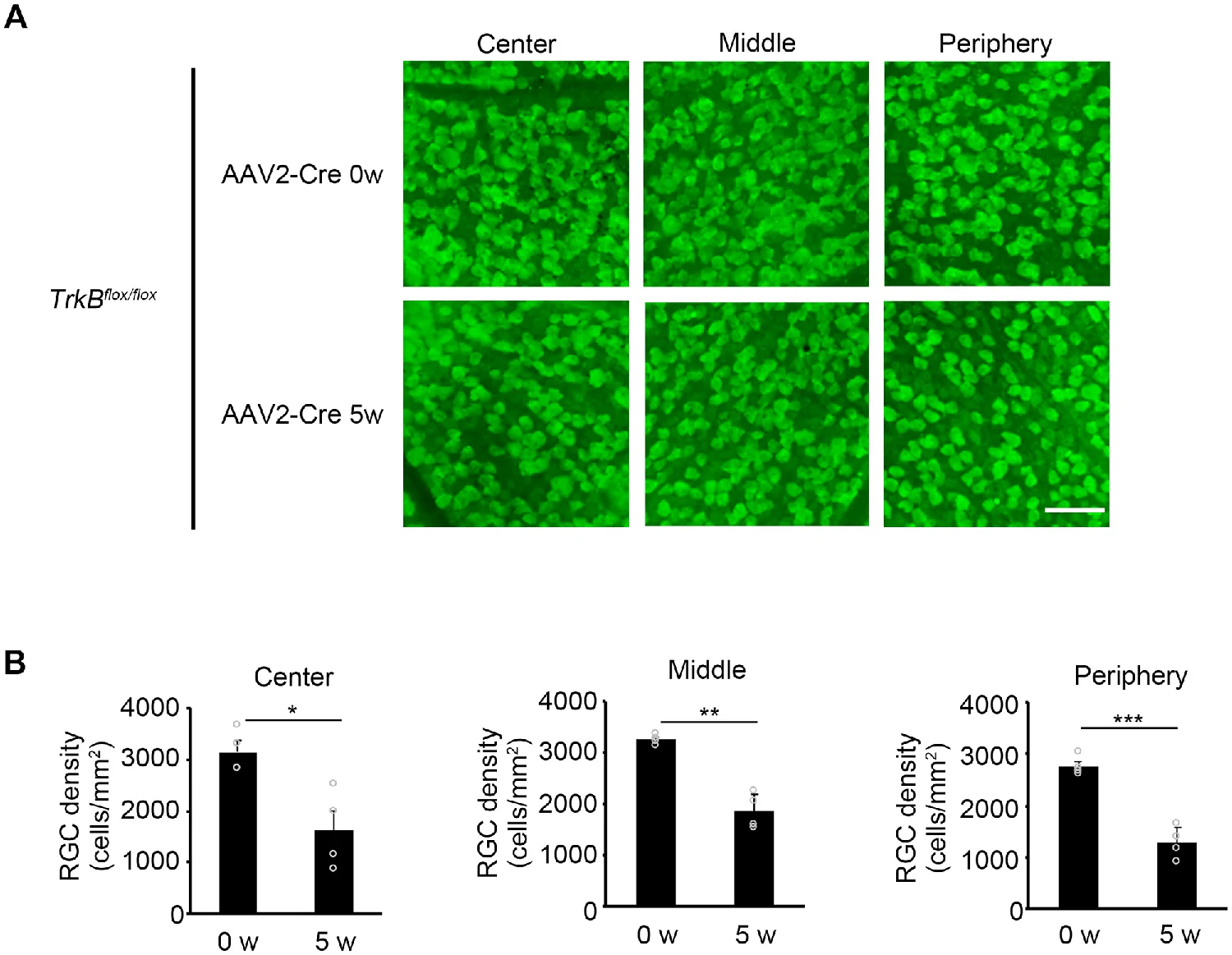
Conditional deletion of TrkB in mature RGCs induces progressive RGC loss. (**A**) Representative images of RBPMS-immunostained retinal flatmounts from TrkB^flox/flox^ mice before AAV2-Cre administration and 5 weeks after administration. Images were acquired from the center, middle, and periphery of the retina. *Scale bar*: 50 µm. (**B**) Quantification of RBPMS-positive RGC density in the center, middle, and periphery of the retina. RGC density was significantly reduced in all retinal regions 5 weeks after AAV2-Cre–mediated TrkB deletion. Data are presented as mean ± SEM. Statistical significance was determined using Welch's unpaired *t*-test (*n* = 4 eyes from 4 mice per group). *P < 0.05, **P < 0.01, ***P < 0.001.

## Discussion

In this study, we investigated the role of TrkB signaling in the maintenance of mature RGCs by inducing acute TrkB deletion in 7-week-old mice. Intravitreally administered AAV2-Cre predominantly transduced RGCs and effectively reduced TrkB levels in the retina of TrkB^flox/flox^ mice. Acute TrkB deletion decreased RGC marker protein levels, reduced retinal responses and RGC density 5 weeks after AAV2-Cre administration, at 12 weeks of age. In contrast, no significant reduction in RGC density was detected in mice with developmental TrkB deletion at 12 weeks of age, although this finding should be interpreted cautiously because of the limited sample size. These findings indicate that TrkB signaling is required for the maintenance of mature RGCs and that acute reduction of TrkB signaling in adulthood can compromise both RGC survival and retinal function.

An important aspect of this study is the difference between acute and developmental TrkB deletion. We previously generated TrkB conditional knockout mice using a c-kit-Cre line, in which TrkB is deleted from early developmental stages in RGCs and amacrine cells.^18^ In the present study, RGC density was not significantly altered in TrkB^flox/flox^;c-kit-Cre mice at 12 weeks of age. By contrast, AAV2-Cre administration to 7-week-old TrkB^flox/flox^ mice induced RGC loss within 5 weeks, at 12 weeks of age. These distinct outcomes may reflect differences in compensatory responses to developmental and acute TrkB deletion. It has been reported that BDNF heterozygous mice are resistant to retinal degeneration, potentially due to compensatory upregulation of other neurotrophic factors.^24^ Similarly, it is possible that developmental loss of TrkB in TrkB^flox/flox^;c-kit-Cre mice induced compensatory changes in other neurotrophic signaling pathways, which may contribute to the apparent preservation of RGCs at this age. In mature RGCs, however, such compensatory mechanisms may be limited, making adult RGCs more vulnerable to acute TrkB reduction.

The present findings may also be relevant to glaucomatous neurodegeneration. Reduced BDNF levels have been reported in patients with normal-tension glaucoma.^25^ Because neurotrophic support in glaucoma is likely to decline during adulthood rather than to be absent from development, acute TrkB deletion may more closely model an adult-onset reduction in BDNF–TrkB signaling than developmental knockout. Our finding that acute TrkB reduction induces both RGC loss and retinal functional impairment suggests that reduced BDNF–TrkB signaling may contribute to RGC vulnerability in glaucoma. However, because the present study directly manipulated TrkB rather than BDNF, further studies are required to clarify the contribution of altered BDNF availability to RGC degeneration.

These findings also support the rationale for TrkB-targeted neuroprotection. We previously showed that membrane-associated intracellular TrkB (iTrkB) activates downstream TrkB signaling and promotes neuroprotection and axon regeneration in experimental models of optic nerve injury and glaucoma.^18^ Further studies are needed to determine whether iTrkB can prevent RGC degeneration caused by acute TrkB loss or glaucomatous stress. However, several limitations of the present study should be considered. Although AAV2-Cre predominantly transduced RGCs, minor effects on other retinal cell types cannot be excluded. In addition, the relatively small sample sizes in some experiments, particularly the analysis of developmental TrkB deletion, limit the strength of the conclusions that can be drawn from these findings. Furthermore, only male mice were used in the present study; therefore, potential sex-dependent differences in the effects of TrkB deletion on RGC survival and retinal function could not be evaluated. Future studies including both male and female mice will be important to determine whether the effects of TrkB loss differ between sexes. Finally, the present study examined relatively early time points under basal conditions rather than in an experimental glaucoma model. Longer-term studies under glaucomatous stress will be required to determine the progression of RGC degeneration and to evaluate the therapeutic potential of TrkB activation.

In conclusion, acute TrkB deletion in the adult retina induces RGC degeneration and retinal dysfunction, whereas no significant reduction in RGC density was detected following developmental TrkB deletion at 12 weeks of age. These findings highlight the importance of endogenous TrkB signaling in mature RGC maintenance and support further investigation of TrkB-targeted strategies for glaucoma and related optic neuropathies.

## Supplementary Material

Supplement 1
