# Supplementary material for: Acute Loss of TrkB Signaling Impairs the Survival of Mature Retinal Ganglion Cells and Retinal Function
Source: Invest Ophthalmol Vis Sci. 2026 Oct 28;67(12):75. doi: 10.1167/iovs.67.12.75 (PMC13722991; doi:10.1167/iovs.67.12.75)
Supplement: Supplementary file 1 [file iovs-67-12-75_s001.docx]

**Supplementary Figure 1. Full-length Western blots corresponding to Figure 3B.**
Full-length, uncropped Western blots of retinal lysates from TrkB flox/flox mice at 3 and 5 weeks after intravitreal administration of AAV2-Cre. Blots were probed for TrkB, RBPMS, Calretinin, glutamine synthetase (GS), Recoverin, and β-actin. For each time point, three independent samples (#1–#3 at 3 weeks and #4–#6 at 5 weeks) are shown, with AAV2-Cre-treated (+) and untreated (−) samples. Molecular weight markers (kDa) are indicated on the left of each blot.
